# Vertebral Ankylosis Is Associated with Reduced Cervical Extensor Muscle Bulk and Increased Fatty Degeneration

**DOI:** 10.3390/jcm15010119

**Published:** 2025-12-24

**Authors:** Junho Song, Austen D. Katz, Alex Ngan, Andrew C. Hecht, Sheeraz A. Qureshi, Sohrab Virk

**Affiliations:** 1 Department of Orthopaedic Surgery, Icahn School of Medicine at Mount Sinai, New York, NY 10029, USA; 2 Department of Orthopaedic Surgery, Northwell Health Long Island Jewish Medical Center, NY 11040, USA; 3 Department of Orthopaedic Surgery, Hospital for Special Surgery, New York, NY 10021, USA

**Keywords:** vertebral ankylosis, cervical spine, muscle health, sarcopenia, ankylosing spondylitis, diffuse idiopathic skeletal hyperostosis, Goutallier, fatty infiltration

## Abstract

**Background/Objectives:** Ankylosing spondylitis and diffuse idiopathic skeletal hyperostosis produce long-segment spinal ankylosis, altered biomechanics, and high fracture risk in the cervical spine. Paraspinal muscle degeneration (“spine-specific sarcopenia”) has been linked to pain, disability, and worse outcomes after cervical spine surgery, but the relationship between vertebral ankylosis and cervical paraspinal muscle health is unknown. We aimed to evaluate the association between vertebral ankylosis and cervical paraspinal muscle health using MRI-based measures of muscle quantity and quality. **Methods:** Adult patients with cervical vertebral ankylosis and available cervical MRI were identified at a single academic center and propensity score-matched 1:1 to patients without ankylosing conditions based on age, sex, body mass index, American Society of Anesthesiologists class, and comorbidity index. Axial T2-weighted images at C2-3 through C7-T1 were used to manually trace bilateral deep extensor and deep flexor muscles to obtain bilateral cross-sectional areas (CSAs) at each level. Extensor fatty infiltration was graded using the Goutallier classification. CSAs and Goutallier grades were compared between the matched groups. **Results:** Compared with matched controls, patients with vertebral ankylosis demonstrated significantly smaller deep extensor CSA at multiple cervical levels and higher Goutallier grades in the lower cervical spine and at the cervicothoracic junction. Deep flexor CSA tended to be smaller in the ankylosis group, but differences did not reach statistical significance. **Conclusions:** Vertebral ankylosis is associated with poorer cervical paraspinal muscle health, characterized by reduced extensor muscle bulk and increased fatty degeneration. These findings support conceptualizing ankylosing spinal conditions as disorders of both bone and muscle and highlight the cervicothoracic extensors as a potential target for risk stratification and rehabilitation strategies.

## 1. Introduction

Vertebral ankylosis is a hallmark of several spinal disorders, most notably ankylosing spondylitis (AS) and diffuse idiopathic skeletal hyperostosis (DISH). These conditions are characterized by progressive ossification of spinal ligaments and intervertebral structures that ultimately produces a rigid, “ankylosed” spine. In the cervical region, this can manifest as fixed kyphosis, loss of physiologic motion, and an increased susceptibility to unstable fractures even after low-energy trauma, with substantial attendant risks of neurologic injury and need for complex reconstruction [1,2]. In DISH, flowing anterior osteophytes and ossification of the anterior longitudinal ligament similarly lead to segmental ankylosis and abnormal cervical alignment, and have been implicated in dysphagia, airway compromise, and difficult surgical and anesthetic management [3,4]. In addition to these well-recognized structural consequences, many patients with ankylosing conditions experience chronic pain, reduced activity, and systemic inflammation that may predispose to generalized and regional muscle wasting [5].

Over the past decade, paraspinal muscle health has emerged as an important determinant of symptoms and outcomes in degenerative spine disease. MRI-based measures such as cross-sectional area and fatty infiltration graded with the Goutallier classification have been used to characterize “paraspinal sarcopenia” in both the cervical and lumbar spine [6,7]. Greater fatty degeneration of cervical paraspinal muscles has been associated with worse neck pain, higher disability scores, and less improvement after anterior cervical discectomy and fusion or laminoplasty, as well as with adverse changes in cervical sagittal alignment [7,8,9,10,11]. These data suggest that paraspinal muscle quality is a potentially modifiable risk factor and a relevant imaging biomarker in cervical spine care.

Despite these observations, the relationship between vertebral ankylosis and cervical paraspinal muscle health remains unclear. Prior sarcopenia studies have largely focused on non-ankylosed degenerative cohorts and have not specifically addressed how AS- or DISH-related ankylosis might influence cervical muscle quantity or fatty infiltration [7,8,11]. Therefore, the purpose of this study was to evaluate the association between vertebral ankylosis and cervical paraspinal muscle health in patients with cervical spine pathology. Using MRI-based measurements, we compared cervical paraspinal muscle cross-sectional area and Goutallier grades between patients with vertebral ankylosis and matched controls without ankylosing conditions. We hypothesized that vertebral ankylosis would be associated with smaller muscle cross-sectional area and greater fatty degeneration.

## 2. Methods

### 2.1. Study Design and Patient Selection

We performed a retrospective cohort study at a single academic medical center. After institutional review board approval (Feinstein Institutes for Medical Research, IRB #21-0881) with waiver of informed consent, we identified consecutive adult patients presenting to spine surgery clinic with a radiographic diagnosis of ankylosing spondylitis (AS) or diffuse idiopathic skeletal hyperostosis (DISH) who had a cervical spine MRI available for review. Vertebral ankylosis was defined as complete bony bridging across the disk space between adjacent vertebral bodies on sagittal imaging, consistent with previously described criteria for ankylosing spinal disorders [1,2,3,4].

From the same imaging database, we identified a pool of potential controls consisting of patients without AS, DISH, or other ankylosing conditions who underwent cervical MRI for degenerative pathology (e.g., cervical stenosis, radiculopathy, or myelopathy) and had no evidence of vertebral ankylosis. Patients with prior cervical spine surgery, neoplasm, active infection, or poor-quality MRI precluding reliable muscle measurements were excluded. Demographic and clinical variables including age, sex, race, ethnicity, body mass index (BMI), American Society of Anesthesiologists (ASA) classification, and age-adjusted Charlson Comorbidity Index (CCI) were abstracted from the electronic medical record for all eligible patients. These variables were used in the propensity score model as described below.

### 2.2. Propensity Score Matching

To reduce confounding when comparing patients with and without vertebral ankylosis, we used propensity score matching to construct a balanced control cohort. A logistic regression model was fit with vertebral ankylosis (AS or DISH) as the dependent variable and age, sex, race, ethnicity, BMI, ASA class, and CCI as independent variables. The resulting propensity score represented the estimated probability of having vertebral ankylosis given the observed covariates. Patients in the ankylosis group were then matched 1:1 to patients in the non-ankylosis pool using nearest-neighbor matching without replacement and a caliper width of 0.2 standard deviations of the logit of the propensity score. Covariate balance between groups before and after matching was assessed using standardized mean differences. All subsequent comparative analyses of muscle measurements were performed in the propensity-matched cohort.

### 2.3. MRI-Based Cervical Paraspinal Muscle Health Measurements

All MRI examinations were performed using standard clinical cervical spine protocols at the study institution. Axial T2-weighted images oriented perpendicular to the disk space were used for muscle analysis. Measurements were obtained at the C2-C3, C3-C4, C4-C5, C5-C6, C6-C7, and C7-T1 disk levels, using the slice closest to the center of the disk. When multiple MRI studies were available for a patient, the most recent study meeting image-quality criteria was selected.

**Figure 1:**
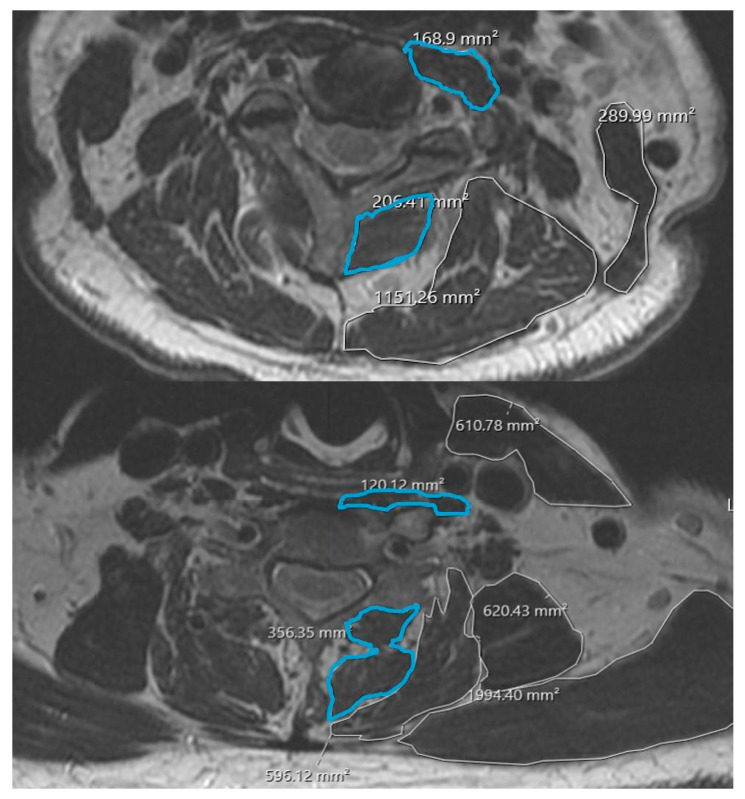
Example cross-sectional area measurements of deep cervical extensors and flexors.

Cervical paraspinal muscle morphology was quantified using the institution’s DICOM viewing software. At each disk level, regions of interest were manually traced around the deep extensor muscles (including multifidus and semispinalis cervicis) and deep flexor muscles (longus colli and longus capitis) bilaterally, following previously described methods for cervical paraspinal assessment [7,8,10]. Cross-sectional area (CSA) for each muscle group was recorded in mm^2^; bilateral values were summed to yield a single CSA measurement per level for deep extensors and deep flexors (Figure 1).

Qualitative muscle quality was graded using the Goutallier classification adapted for cervical paraspinal muscles, which categorizes fatty infiltration on a 0–4 scale based on the proportion of intramuscular fat relative to muscle tissue (Table 1) [6,7]. A Goutallier grade was assigned to the deep extensor compartment at each disk level. All measurements and grades were performed independently by two observers who were blinded to ankylosis status. Interobserver reliability for CSA measurements and Goutallier grades was assessed using the intraclass correlation coefficient (ICC), which demonstrated excellent (>0.75) agreement between observers.

### 2.4. Statistical Analysis

Continuous variables were summarized as mean ± standard deviation and categorical variables as counts and percentages. Baseline demographic and clinical characteristics were compared between propensity score matched groups using paired *t*-tests for continuous variables and chi-square or Fisher’s exact tests for categorical variables, as appropriate.

After propensity score matching, deep extensor CSA, deep flexor CSA, and Goutallier grade at each cervical level were compared between the ankylosis and control groups using paired or independent-samples *t*-tests, depending on distributional assumptions. Given the exploratory nature of the study and limited sample size, *p*-values were not adjusted for multiple comparisons. A two-sided *p* < 0.05 was considered statistically significant. All statistical analyses were performed using SPSS (version 28, IBM, Armonk, New York, NY, USA).

## 3. Results

### 3.1. Patient Cohort and Baseline Characteristics

A total of 34 patients were included, comprising 17 patients with vertebral ankylosis (AS or DISH) and 17 propensity score-matched controls without ankylosing conditions. In the matched cohort, age, sex, BMI, ASA class, and age-adjusted CCI were well balanced between groups. The mean age of the total cohort was 74.2 ± 14.7 years (75.5 ± 15.8 in the ankylosis group vs. 73.0 ± 14.1 in controls, *p* = 0.679). Both cohorts had the same proportion of female patients (35.3%). Distributions of Black race (17.6% vs. 23.5%, *p* = 0.658) and Hispanic ethnicity (23.5% vs. 11.8%, *p* = 0.645) did not differ significantly. Mean BMI was 27.4 ± 6.7 kg/m^2^ in the ankylosis group and 25.5 ± 5.0 kg/m^2^ in controls (*p* = 0.431). Age-adjusted CCI (3.4 ± 1.1 vs. 3.3 ± 1.3, *p* = 0.873) and the proportion of patients with ASA grade ≥ 3 (11.8% vs. 11.8%, *p* = 1.000) were also similar between groups (Table 2).

### 3.2. Cervical Paraspinal Muscle Cross-Sectional Area

Across the cervical spine, patients with vertebral ankylosis demonstrated smaller deep extensor CSA compared with matched controls at multiple levels (Table 3). At C2-C3, deep extensor CSA was 351.3 ± 173.2 mm^2^ in the ankylosis group versus 590.2 ± 465.3 mm^2^ in controls (*p* = 0.048). Similar patterns were observed at mid- and lower-cervical levels: 327.6 ± 117.1 mm^2^ versus 572.4 ± 371.9 mm^2^ at C4-C5 (*p* = 0.027), 360.6 ± 144.7 mm^2^ versus 554.0 ± 349.0 mm^2^ at C5-C6 (*p* = 0.039), and 441.1 ± 257.0 mm^2^ versus 644.7 ± 391.8 mm^2^ at C6-C7 (*p* = 0.033). Differences at C3-C4 (333.8 ± 151.0 vs. 527.6 ± 417.4 mm^2^, *p* = 0.070) and C7-T1 (453.2 ± 220.5 vs. 612.5 ± 304.2 mm^2^, *p* = 0.063) trended toward smaller extensor CSA in the ankylosis group but did not reach statistical significance.

Deep flexor CSA was consistently lower in the ankylosis cohort, although these differences did not achieve statistical significance at any level. Deep flexor CSA was 148.4 ± 102.2 mm^2^ versus 181.8 ± 67.0 mm^2^ at C2-C3 (*p* = 0.167), 132.2 ± 54.6 mm^2^ versus 172.4 ± 92.3 mm^2^ at C3-C4 (*p* = 0.183), and 129.5 ± 54.2 mm^2^ versus 184.1 ± 104.6 mm^2^ at C7-T1 (*p* = 0.108), with similar non-significant trends at intermediate levels.

### 3.3. Cervical Paraspinal Muscle Quality (Goutallier Grade)

Vertebral ankylosis was associated with greater fatty degeneration of the cervical paraspinal extensors at selected levels. At C5-C6, mean Goutallier grade was 2.92 ± 0.86 in the ankylosis group compared with 1.85 ± 0.69 in controls (*p* < 0.001). At C7-T1, Goutallier grade was likewise higher in the ankylosis group (3.23 ± 0.60 vs. 2.12 ± 0.86, *p* < 0.001). At more cephalad levels, Goutallier grades were higher but not significantly different between groups: 2.54 ± 0.88 versus 2.08 ± 1.19 at C2-C3 (*p* = 0.135), 3.00 ± 0.71 versus 2.43 ± 0.92 at C3-C4 (*p* = 0.107), 2.54 ± 0.88 versus 2.38 ± 0.73 at C4-C5 (*p* = 0.327), and 2.69 ± 0.95 versus 2.20 ± 0.78 at C6-C7 (*p* = 0.113).

Collectively, these findings indicate that vertebral ankylosis is associated with smaller cervical deep extensor muscle CSA at multiple levels and with more advanced fatty infiltration in the lower cervical and cervicothoracic junction.

## 4. Discussion

The present study demonstrates that vertebral ankylosis is associated with objectively poorer cervical paraspinal muscle health in a matched cohort of patients with cervical spine pathology. Compared with propensity score-matched controls, patients with ankylosing conditions had significantly smaller deep extensor CSA at multiple cervical levels and higher Goutallier grades in the lower cervical spine and at the cervicothoracic junction. Deep flexor CSA also tended to be smaller in the ankylosis group, although these differences did not reach statistical significance. Together, these findings support the concept that vertebral ankylosis may be linked not only to altered bony architecture but also to concomitant deterioration of the surrounding paraspinal musculature.

Prior work in ankylosing spondylitis (AS) and diffuse idiopathic skeletal hyperostosis (DISH) has focused predominantly on fracture risk, deformity, and perioperative outcomes in the setting of a rigid spine [1,2,3,4,12,13]. These studies highlight how long-segment ankylosis converts the spine into a lever arm that is prone to catastrophic failure, particularly in the cervical region. At the same time, systemic inflammation, pain, and reduced mobility in AS and DISH have been linked to generalized sarcopenia and changes in body composition [5,14,15,16]. Our findings extend this literature by showing that patients with vertebral ankylosis may exhibit localized paraspinal sarcopenia of the cervical extensors, as reflected by both reduced CSA and increased fatty degeneration.

There is growing recognition that paraspinal muscle quality is a key determinant of symptoms and surgical outcomes in degenerative cervical disease. Increased fatty infiltration of cervical paraspinal muscles, graded using the Goutallier classification, has been associated with worse neck pain, higher disability scores, less improvement after anterior cervical discectomy and fusion or laminoplasty, and adverse changes in cervical sagittal alignment [7,8,9,10,11]. Similar relationships between diminished muscle CSA, higher Goutallier grade, and worse functional status have been described in the lumbar spine, including evidence that prior surgery and chronic pathology are associated with smaller paralumbar muscles and more fatty degeneration [6,17,18,19,20,21]. Within this context, our data suggest that vertebral ankylosis may identify a subgroup of patients at particularly high risk for poor cervical paraspinal muscle quality, especially in the lower cervical and cervicothoracic segments. This regional pattern is biomechanically plausible. In ankylosing conditions, long segments of fused vertebrae behave as a single rigid lever arm, shifting motion and mechanical stress to the remaining mobile junctional levels, most notably the lower cervical spine and cervicothoracic junction. Prior work in ankylosing spondylitis and DISH has highlighted this area as a common site of fracture, deformity, and sagittal malalignment, reflecting high local mechanical demand [1,2,12,13]. Chronic overload of the posterior tension band, in combination with pain, limited mobility, and systemic inflammation, may promote selective atrophy and fatty degeneration of the extensor musculature at these junctional levels, which is consistent with our findings and with broader literature on paraspinal sarcopenia in deformity and degenerative cohorts [6,17,18].

The pattern we observed—more pronounced differences in the deep extensors than in the deep flexors, and greater fatty infiltration at C5-C6 and C7-T1—may have both biomechanical and clinical relevance. Extensors play a dominant role in maintaining cervical lordosis and horizontal gaze; impairment of this “posterior tension band” could contribute to fixed chin-on-chest deformity, difficulty compensating for sagittal malalignment, and neck fatigue in patients with ankylosing conditions [1,2]. The cervicothoracic junction is also a transitional zone between ankylosed and more mobile segments in many AS/DISH patients, potentially concentrating stress and accelerating muscle degeneration in this region. These mechanisms may help explain why ankylosed spines are vulnerable not only to fracture and deformity but also to functional limitations related to muscle insufficiency.

Clinically, our findings reinforce the value of incorporating paraspinal muscle assessment into the evaluation of patients with ankylosing cervical pathology. Routine review of cervical MRI for paraspinal CSA and Goutallier grade can provide additional information beyond bony alignment and canal compromise and may help identify patients who could benefit from targeted prehabilitation or postoperative rehabilitation focused on extensor conditioning. In surgical candidates, awareness of underlying paraspinal sarcopenia may influence expectations for postoperative pain, disability, and sagittal balance, and might inform decisions about fusion length and the need for more rigid constructs [6,7,8,9,10,11]. In addition, prior work in ankylosing spondylitis and axial spondyloarthritis has shown that structured exercise and posture/extension programs can improve spinal mobility, disease activity, and physical function, and may favorably influence trunk muscle performance and morphology [22,23]. Incorporating targeted cervical extensor and postural training into rehabilitation protocols for patients with vertebral ankylosis therefore represents a biologically plausible and testable strategy, and our findings provide a rationale for future interventional studies examining whether such programs can mitigate paraspinal degeneration or improve clinical outcomes in this high-risk group. From a research standpoint, vertebral ankylosis could serve as a useful stratification variable or effect modifier in future outcome studies that incorporate muscle parameters.

This study has several limitations. Its retrospective, single-center design and small sample size limit generalizability and preclude detailed subgroup analyses of AS versus DISH or different patterns of ankylosis. The cross-sectional design does not allow us to determine whether ankylosis precedes or accelerates paraspinal muscle degeneration, or whether poor muscle health contributes to the development of ankylosis. We relied on imaging-based measures of muscle quantity and quality without concurrent, uniformly collected functional or patient-reported outcome data, so we cannot directly link the observed differences to clinical symptoms in this cohort. Additionally, the muscle measurements were based on two-dimensional CSA obtained at discrete axial levels, which is sensitive to slice position and level selection and may not fully capture global muscle volume or the spatial distribution of fatty infiltration; more advanced three-dimensional muscle volumetry and quantitative fat metrics (e.g., MRI-based fat fraction) were not available. Future studies incorporating 3D MRI-based muscle volume and quantitative fat-fraction techniques would help validate and strengthen these findings. Although propensity score matching reduced measurable confounding, unmeasured factors such as physical activity level, medication exposure, and duration of disease may still differ between groups. Finally, multiple comparisons were not adjusted given the exploratory nature of the analysis.

Despite these limitations, the present study adds to the growing body of literature on spine-specific sarcopenia by demonstrating that vertebral ankylosis is associated with smaller cervical extensor CSA and greater fatty degeneration on MRI [14,15,16,18,24]. These findings suggest that ankylosing spinal conditions should be conceptualized as disorders of both bone and muscle, and they highlight the cervicothoracic extensor musculature as a potential focus for future studies and rehabilitation strategies.

## 5. Conclusions

Patients with vertebral ankylosing conditions demonstrated smaller deep extensor cross-sectional area at multiple cervical levels and greater fatty degeneration. These findings suggest that ankylosing spinal disorders involve not only rigid, fused segments but also concomitant deterioration of the surrounding musculature. Incorporating routine assessment of paraspinal muscle quantity and quality into imaging review may improve risk stratification and help guide expectations and rehabilitation strategies. Future work should clarify the longitudinal relationship between ankylosis and paraspinal degeneration and determine whether targeted interventions to preserve or restore cervical extensor muscle health can modify outcomes in this high-risk population.

## Figures and Tables

**Table 1:** Goutallier Grading System of Muscle Fatty Infiltration.

Stage	Muscle Description
0	No fatty streak in muscle
1	Fatty streak present in muscle
2	Less fat than muscle (fat < muscle)
3	Equal amounts of fat and muscle (fat = muscle)
4	More fat than muscle (fat > muscle)

**Table 2:** Patient Characteristics.

	Total Cohort	Ankylosis	Control	*p*-Value
Total *N*	34	17	17	
Age	74.2 ± 14.7	75.5 ± 15.8	73.0 ± 14.1	0.679
Female Sex	12 (35.3%)	6 (35.3%)	6 (35.3%)	1.000
Black Race	7 (20.6%)	3 (17.6%)	4 (23.5%)	0.658
Hispanic Ethnicity	6 (17.6%)	4 (23.5%)	2 (11.8%)	0.645
BMI	26.4 ± 5.9	27.4 ± 6.7	25.5 ± 5.0	0.431
Age-adjusted CCI	3.3 ± 1.2	3.4 ± 1.1	3.3 ± 1.3	0.873
ASA ≥ 3	4 (11.8%)	2 (11.8%)	2 (11.8%)	1.000

BMI, body mass index. CCI, Charlson Comorbidity Index. ASA, American Society of Anesthesiologists.

**Table 3:** Cervical Paraspinal Muscle Health in Patients With and Without Vertebral Ankylosis.

Muscle Health Parameter	Ankylosis (*N* = 17)	Control (*N* = 17)	*p*-Value
**C2-C3**			
Deep Extensor CSA	351.3 ± 173.2	590.2 ± 465.3	**0.048**
Deep Flexor CSA	148.4 ± 102.2	181.8 ± 67.0	0.167
Goutallier Grade	2.54 ± 0.88	2.08 ± 1.19	0.135
**C3-C4**			
Deep Extensor CSA	333.8 ± 151.0	527.6 ± 417.4	0.070
Deep Flexor CSA	132.2 ± 54.6	172.4 ± 92.3	0.183
Goutallier Grade	3.00 ± 0.71	2.43 ± 0.92	0.107
**C4-C5**			
Deep Extensor CSA	327.6 ± 117.1	572.4 ± 371.9	**0.027**
Deep Flexor CSA	137.4 ± 49.9	178.0 ± 73.8	0.132
Goutallier Grade	2.54 ± 0.88	2.38 ± 0.73	0.327
**C5-C6**			
Deep Extensor CSA	360.6 ± 144.7	554.0 ± 349.0	**0.039**
Deep Flexor CSA	123.9 ± 42.9	170.6 ± 136.7	0.126
Goutallier Grade	2.92 ± 0.86	1.85 ± 0.69	**<0.001**
**C6-C7**			
Deep Extensor CSA	441.1 ± 257.0	644.7 ± 391.8	**0.033**
Deep Flexor CSA	125.4 ± 41.8	189.5 ± 122.9	0.094
Goutallier Grade	2.69 ± 0.95	2.20 ± 0.78	0.113
**C7-T1**			
Deep Extensor CSA	453.2 ± 220.5	612.5 ± 304.2	0.063
Deep Flexor CSA	129.5 ± 54.2	184.1 ± 104.6	0.108
Goutallier Grade	3.23 ± 0.60	2.12 ± 0.86	**<0.001**

Bold values indicate statistical significance (*p* < 0.05). CSA, cross-sectional area (mm^2^).

## Data Availability

The data presented in this study are available on request from the corresponding author. They are not publicly available as they were derived from individual patients and electronic medical chart review at our institution.
